# Delayed postoperative hemorrhage (DEPOH) in an Irish Wolfhound with the *SERPINF2* c.605 T/T genotype: case description and variant prevalence across dog breeds

**DOI:** 10.3389/fvets.2025.1609780

**Published:** 2025-11-06

**Authors:** Michael H. Court, Melissa Hardy, Keith R. Forbes, Hong Yang, Tania Perez Jimenez

**Affiliations:** 1 Program in Individualized Medicine, Pharmacogenomics Laboratory, Department of Veterinary Clinical Sciences, College of Veterinary Medicine, Washington State University, Pullman, WA, United States; 2 Paws of Pleasanton Animal Hospital, Pleasanton, CA, United States; 3 Division of Animal Industry, Nevada Department of Agriculture, Sparks, NV, United States

**Keywords:** delayed postoperative hemorrhage, hyperfibrinolysis, greyhound, Scottish Deerhound, Irish Wolfhound

## Abstract

**Introduction:**

Delayed postoperative hemorrhage (DEPOH) is a life-threatening complication of major surgical procedures in Greyhounds affecting up to 26–30% of dogs. DEPOH has also been reported in Scottish Deerhounds, but it is unclear whether any other breeds are affected. A genetic variant (c.605 C > T) was recently discovered in the *SERPINF2* gene that is currently used as a biomarker for increased risk of DEPOH. The objective of this study was to provide the first report of DEPOH occurring in association with the *SERPINF2* variant in an Irish Wolfhound. *SERPINF2* variant prevalence was also surveyed across dog breeds to identify other breeds that may be at increased risk for DEPOH.

**Methods:**

Case history and medical records from the affected dog were reviewed. *SERPINF2* genotypes were determined for this dog and for 63 different breeds (including 16 different sighthound breeds) using DNA samples from 4,044 pet dogs supplemented with publicly available genotype data.

**Results:**

The reported case was a 2-year-old healthy female Irish Wolfhound who underwent ovariohysterectomy and prophylactic gastropexy procedures without complication. Two days later extensive bruising was noted on the ventral abdomen that spread to the torso by day 4 when the dog collapsed and died. Necropsy revealed extensive external and internal bruising with free and clotted blood in the abdomen. All ligatures were intact, and no sources of bleeding were identified. Subsequent genotyping indicated that this case was homozygous for the *SERPINF2* T/T high risk genotype. Greyhounds had the highest T/T genotype prevalence (29%), Irish Wolfhounds had the second highest prevalence (24%), and Scottish Deerhounds had the 8th highest prevalence (6%). Nine of 16 sighthound breeds had a T/T prevalence of at least 5%, while none of 47 non-sighthound breeds had a prevalence this high. Four non-sighthound breeds, including Shetland sheepdog, Newfoundland, English bulldog and French bulldog had a carrier (C/T or T/T) genotype prevalence over 20%.

**Discussion:**

These results suggest that DEPOH could occur in most, but not all sighthound breeds. Some non-sighthound breeds might also be susceptible.

## 1 Introduction

Delayed postoperative hemorrhage (DEPOH) is a breed-associated life-threatening complication of major surgical procedures in dogs (1–4). This bleeding disorder was originally described in Greyhounds, affecting as many as 26–30% of dogs undergoing various surgical procedures, including orchiectomy and ovariohysterectomy (4). DEPOH typically presents as unexpected or excessive bleeding first detected hours to days after a major surgery, dental procedure, or other trauma. Bleeding may first appear as peri-incisional bruising that becomes more generalized; seeping or frank bleeding from the incision; or undetected internal bleeding from the surgical site.

The primary defect in DEPOH is hypothesized to be hyperfibrinolysis resulting in weaker blood clots and premature breakdown of these clots (5–7). Routinely used assays for primary and secondary hemostatic defects, such as prothrombin time, activated partial thromboplastin time, blood platelet count, and Von Willebrand factor yield results within the reference range (4). Diagnosis is usually based on exclusion of other causes and response to treatment with antifibrinolytic drugs such as aminocaproic acid and tranexamic acid.

DEPOH also occurs in another sighthound dog breed, the Scottish Deerhound. A recent case–control genome-wide association study involving 269 Scottish Deerhounds identified a genetic variant (c.605 C > T) in the *SERPINF2* gene associated with DEPOH risk (8). This gene encodes alpha-2 antiplasmin, which is the primary negative regulator of fibrinolysis by plasmin. Congenital alpha-2 antiplasmin deficiency (complete or partial) is a rare genetic disorder in people that manifests as delayed bleeding after trauma, surgery, or dental procedures (9, 10). Although the *SERPINF2* c.605 C > T variant is predicted to change the alpha-2 antiplasmin protein sequence (alanine to valine at position 202), the functional consequence of this change on alpha-2 antiplasmin function has not been determined.

To date, case reports of DEPOH have not been published for breeds other than Greyhounds or Scottish Deerhounds. Interestingly, a recent study using a point-of-care whole blood viscoelastic monitoring device demonstrated weaker blood clots and evidence for hyperfibrinolysis in 27 healthy Irish Wolfhounds when compared with 27 age-matched healthy large-breed control dogs (11). These results (weaker blood clots and hyperfibrinolysis) were similar to those previously reported using the same viscoelastic monitor in 53 healthy Greyhounds compared with 38 non-Greyhound dogs (5). Taken together, these results suggest that Irish Wolfhound dogs may be susceptible to DEPOH, like Greyhounds and Scottish Deerhounds. Furthermore, given the high genetic relatedness between these 3 sighthound breeds (12), it is possible that these breeds share the same genetic variant (*SERPINF2* c.605 C > T) associated with DEPOH in Scottish Deerhounds.

Here we report a case of suspected DEPOH in an Irish Wolfhound that was submitted to the Comparative Pharmacogenomics laboratory for *SERPINF2* c.605 C > T genotyping. *SERPINF2* variant prevalence was also surveyed across dog breeds to identify other breeds that may be at increased risk for DEPOH.

## 2 Materials and methods

### 2.1 DEPOH case description

This case involved a 2-year-old 46 kg healthy female Irish Wolfhound who received routine ovariohysterectomy and prophylactic gastropexy surgical procedures. Medical history was unremarkable other than experiencing a small ear laceration from a dog bite at 7 months of age. This was initially treated conservatively by bandaging but required surgical repair by suturing 1 week after the initial trauma because of persistent bleeding from the wound. No abnormalities were detected on a routine physical examination prior to the current surgery. Presurgical hematology and chemistry panel values were within the reference ranges for the laboratory other than a slightly decreased white blood cell count (4.6 × 10^3^ / μL, reference range 4.9–17.7 × 10^3^ / μL) and a slightly increased hematocrit (57.5%, reference range 38.3–56.5%).

The dog was premedicated with hydromorphone (0.15 mg kg^−1^, SC), maropitant (1 mg kg^−1^, SC), and midazolam (0.2 mg kg^−1^, IV), and anesthesia induced with propofol (4 mg kg^−1^, IV). The trachea was intubated, and anesthesia maintained with isoflurane in oxygen. Carprofen (2.2 mg kg^−1^, SC) was administered 30 min after induction. The dog was placed in dorsal recumbency and the abdominal skin clipped and then disinfected using chlorhexidine scrub and solution.

The gastropexy was performed through a 20 cm incision caudal to xyphoid to mid abdomen. A 1.5 cm incision was made through the serosa and initial muscle layer of the 10th rib midway between dorsum and ventrum. A matching 1.5 cm incision was made in the serosa of the cranial surface of the stomach near the greater curvature, avoiding superficial blood vessels. The cranial margin of the stomach incision was sutured to the cranial margin of the body wall incision, and the caudal margin of the stomach incision was sutured to the caudal margin of the body wall incision, each with 3–0 poliglecaprone 25 absorbable monofilament sutures, in a simple continuous pattern.

The ovariohysterectomy was performed next. The uterus was isolated and the left ovary was exteriorized for visualization after stretching of the suspensory ligament. Two hemostats were placed across the ovarian pedicle. The clamp farthest from the ovary was then replaced with a size 2–0 poliglecaprone monofilament ligature tied using a Miller’s knot. The pedicle was then transected below the remaining clamp and checked for evidence of bleeding before release. The same technique was used for the right ovary. The cervix was then exposed, and a similar 2 clamp technique was used on the uterine body proximal to the cervix to place a size 2–0 poliglecaprone monofilament ligature tied with a Miller’s knot. The uterine body was transected below the clamp and the stump inspected for bleeding when returned to a normal anatomic position. After inspecting the abdomen for signs of hemorrhage, the linea alba was closed using size 0 polydioxanone sutures in a cruciate pattern. The subcutaneous tissues and skin were closed using 2–0 poliglecaprone monofilament sutures.

**Figure 1:**
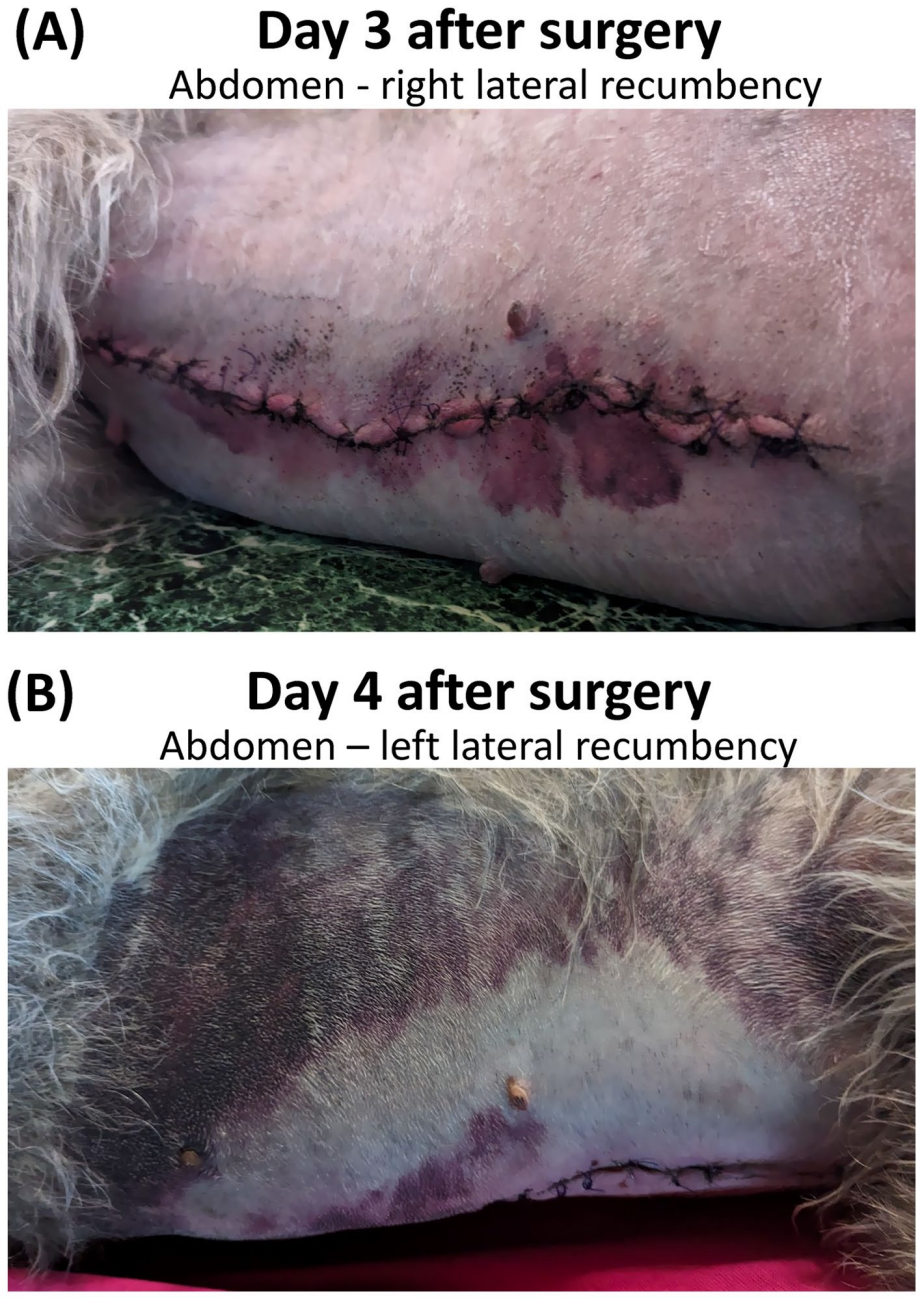
Extensive progressive peri-incisional bruising on the ventral abdomen of an Irish Wolfhound on day 3 **(A)** and day 4 **(B)** following surgical ovariohysterectomy and prophylactic gastropexy. Photographic images provided by owner with permission.

Recovery from anesthesia was unremarkable. The dog was discharged to the owner that evening with instructions for post-surgical care, including administration of carprofen tablets (2.2 mg kg^−1^ orally) and gabapentin capsules (4.4 mg kg^−1^ orally) every 12 h. For the first 2 days after surgery the owner reported that the dog was relatively quiet and appeared somewhat depressed, which was attributed to the sedative effects of gabapentin. Although the dog was much brighter on Day 3, the owner noted extensive purple discoloration of the skin (purpura) on the ventral abdomen (Figure 1A) that spread to the torso on Day 4 (Figure 1B). On the evening of Day 4, the dog was observed by the owner to collapse, struggle momentarily as if experiencing a seizure, and died. The owner brought the dog to an emergency clinic and requested a necropsy.

A gross necropsy examination was performed by the Nevada Department of Agriculture Animal Disease Laboratory. Major findings included significant skin discoloration (dark red/purple) over the entire ventral abdomen and thorax. Extensive subcutaneous hemorrhages up to 3 cm deep on the cut surface were noted in the same areas. Visible hemorrhages were also observed on the pleural surfaces of the thoracic wall, the serosal surface of the lower trachea, epicardial surface of the heart, the serosal surface of the urinary bladder, and scattered throughout the mesentery. The abdominal cavity contained 100 mL of free blood and a large (41 gram) blood clot. The stomach was sutured to the left abdominal wall, which was consistent with a prophylactic gastropexy. All ligatures were intact and secure on both ovarian pedicles and the uterine stump. Samples were collected from liver, heart and lungs for aerobic bacterial culture. No pathogens were subsequently identified. The pathologist suspected DEPOH and a sample of liver was submitted for *SERPINF2* c.605 C > T genotyping.

### 2.2 *SERPINF2* c.605 C > T genotyping

A custom allele discrimination assay (Applied Biosystems TaqMan SNP Genotyping Assay, Thermo Fisher Scientific, Waltham MA) was used to genotype DNA samples for the *SERPINF2* c.605 C > T gene variant as previously described in detail (8). Briefly, primers and probes were 5’-ACG CTG CGG AGG TTA GAG-3′ (forward primer), 5’-CCC AGG TCC TGG CAA AGG-3′ (reverse primer), 5’-CCA GAG TCT GCA TGC AG-3′ (C-allele probe labeled with VIC dye), and 5’-CCA GAG TCT ACA TGC AG-3′ (T-allele probe labeled with FAM dye). Assays were performed according to the manufacturer’s directions using a real-time PCR instrument (CFX96 Touch, Bio-Rad, Hercules, CA). The PCR method involved an initial denaturation step at 95 °C for 10 min, and 55 cycles of 95°C for 15 s and 60 °C for 90 s. Genotype calls were made using the CFX90 allele discrimination function. Positive and negative controls were included with each run. Positive control samples were randomly selected dog DNA samples with known *SERPINF2* c.605 C > T genotype (C/C, C/T and T/T). The negative control sample was DNA-free pure water.

### 2.3 Dog breed DNA sampling

Stored DNA samples (*N* = 4,044) from client-owned dogs were retrieved from the Washington State University Veterinary Teaching Hospital Patient DNA Bank and the Comparative Pharmacogenomics Laboratory Sighthound DNA Bank. DNA had been extracted from buccal swabs or whole blood samples provided by the dog’s owner. Hospital patient samples were from dogs living in the Pacific Northwest of the United States, while the Sighthound DNA bank samples were obtained primarily by mail from dogs living throughout the United States. The dog’s breed was based on breed registration information (if available) and by owner designation. The 4,044 DNA samples represented 63 different dog breeds (16 Sighthound breeds and 47 other breeds) with a minimum of 20 DNA samples per breed and 186 DNA samples from mixed-breed dogs. The designation of a breed as belonging to the Sighthound group was primarily based on the breed inclusion criteria used for Lure Coursing established by the American Kennel Club^1^. The collection, storage, and use of the DNA samples used in this study were approved by the Institutional Animal Care and Use Committee at Washington State University (protocols #04194 and #04539).

### 2.4 Retrieval of publicly available *SERPINF2* c.605 C > T genotype data

*SERPINF2* c.605 C > T genotype data were extracted from publicly available whole genome sequences for 315 unrelated dogs from 50 different dog breeds (13). *SERPINF2* c.605 C > T genotypes were also obtained from the same database for 27 unrelated gray wolves (*Canis lupus*) sampled from populations in North America, Europe and Asia.

### 2.5 Polyphen-2 analysis

The online tool PolyPhen-2 (Polymorphism Phenotyping v2)^2^, was used to evaluate the possible functional impact of the *SERPINF2* c.605 C > T variant, which results in an alanine to valine substitution at position 202 in the canine alpha-2 antiplasmin polypeptide. For this analysis, the reference canine alpha-2 antiplasmin amino acid sequence (XP_005624992.1) was retrieved from the National Center for Biotechnology Information protein sequence repository^3^.

## 3 Results

### 3.1 *SERPINF2* c.605 C > T genotype of the DEPOH case

*SERPINF2* c.605 C > T genotyping indicated that the DEPOH case had the *SERPINF2* c.605 T/T genotype (homozygous variant).

### 3.2 *SERPINF2* c.605 C > T variant prevalence in sighthound and non-sighthound breeds

Canine population genotype frequencies and breed-specific prevalence of the *SERPINF2* c.605 C > T variant were determined by genotyping DNA samples collected from 63 different breeds and supplemented using publicly available dog whole genome sequence data (13). At least 20 dogs per breed were sampled. Breeds evaluated included 16 sighthound breeds, 47 other (non-sighthound) dog breeds, and 186 mixed-breed dogs (4,359 dogs genotyped in total). *SERPINF2* c.605 C > T genotype data for 27 gray wolves sampled from geographically diverse populations were also obtained from the same public database (13) to determine the ancestral origin of this variant.

**Figure 2:**
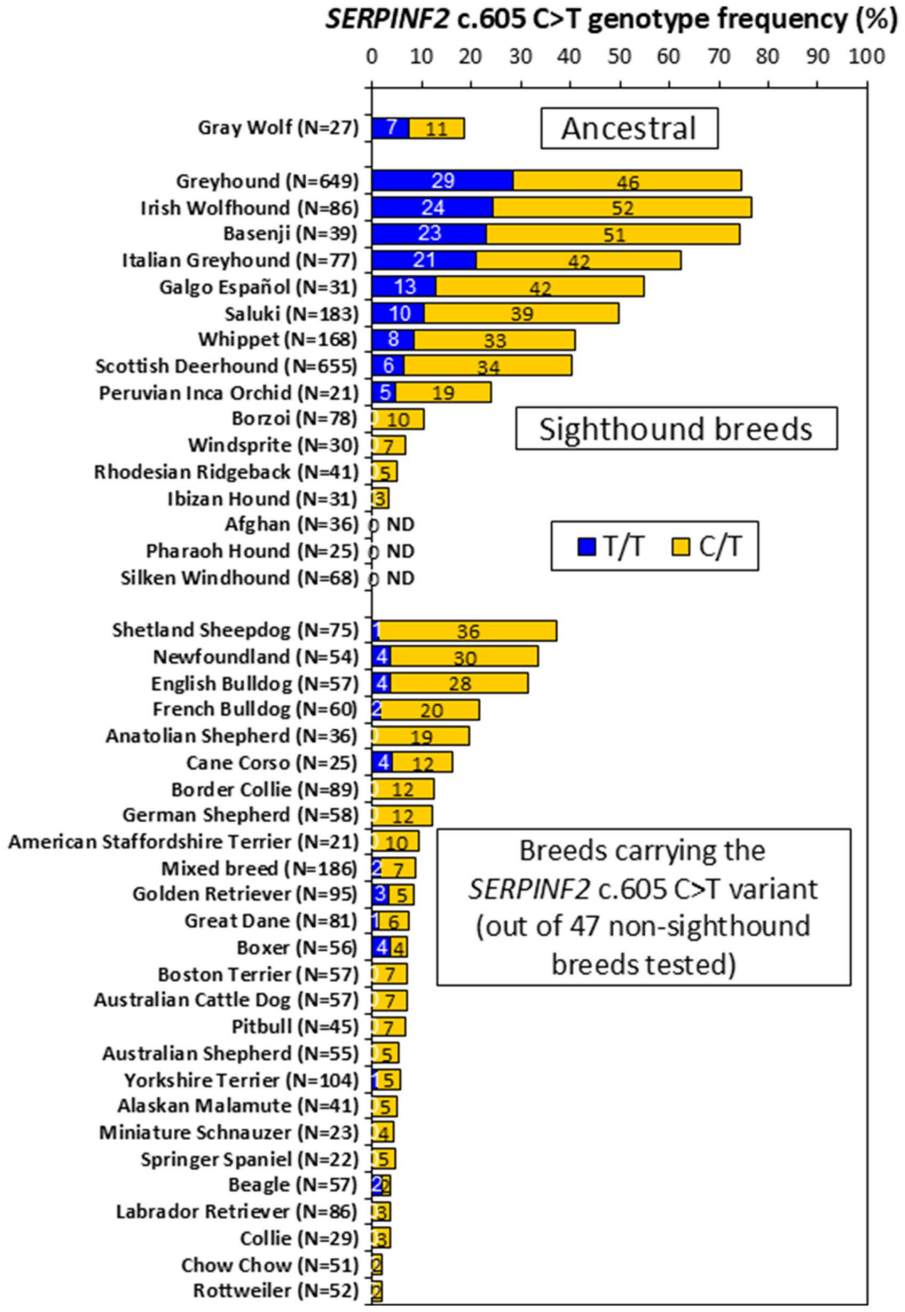
Prevalence of *SERPINF2* c.606 C > T genotypes in 16 sighthound breeds, 47 non-sighthound breeds, mixed breed dogs, and gray wolves. Each bar shows the homozygous variant (T/T - dark blue) and heterozygous (T/C - gold) genotype frequencies for each group. *N* – number of canines tested. ND – variant not detected. Non-sighthound dog breeds that were tested but lacked the *SERPINF2* variant included Bernese Mountain Dog (*N* = 46); Brittany Spaniel (*N* = 27); Cardigan Welsh Corgi (*N* = 22); Cavalier King Charles Spaniel (*N* = 27); Chihuahua (*N* = 23); Cocker Spaniel (*N* = 32); Dachshund (*N* = 25); Doberman Pinscher (*N* = 41); Miniature Dachshund (*N* = 20); Miniature Poodle (*N* = 22); Old English Sheepdog (*N* = 22); Pembroke Welsh Corgi (*N* = 24); Pomeranian (*N* = 21); Pug (*N* = 25); Saint Bernard (*N* = 25); Samoyed (*N* = 24); Shih Tzu (*N* = 21); Siberian Husky (*N* = 25); Soft-coated Wheaten Terrier (*N* = 30); Standard Poodle (*N* = 25); Toy Poodle (*N* = 20); and Weimaraner (*N* = 22).

Supplementary Table 1 provides the numbers of individual animals with each *SERPINF2* c.605 genotype (C/C, C/T or T/T) as well as the variant allele frequencies (number of variant T alleles expressed as a percentage of the total number of alleles) for each dog breed, mixed-breed dogs and wolves. Figure 2 shows the percentage of canines within each group with the *SERPINF2* c.605 homozygous variant (T/T) genotype and the heterozygous variant (C/T) genotype (8).

Greyhounds had the highest variant (T) allele frequency (52%) of all dog breeds evaluated with a high-risk T/T genotype prevalence of 29%. Irish Wolfhound (51%), Basenji (49%) and Italian Greyhound (42%) breeds also showed a high allele prevalence (frequencies over 35%). T/T genotype prevalence in these breeds ranged from 21 to 24%. Galgo Español (34%), Saluki (30%) Whippet (25%), Scottish Deerhound (23%), and Peruvian Inca Orchid (14%), showed a moderate allele prevalence (frequencies between 10 and 35%). T/T genotype prevalence in these breeds ranged from 5 to 13%. Borzoi (5%), Windsprite (3%), Rhodesian Ridgeback (2%), and Ibizan Hound (2%) showed a low allele prevalence (frequencies less than 10%). None of the dogs tested in these breeds had the T/T genotype. The *SERPINF2* c.605 T variant allele was not detected in any Afghan, Pharaoh Hound, or Silken Windhound dog tested.

The *SERPINF2* c.605 T variant allele was found in other (non-sighthound) dog breeds, although none of those tested showed an allele prevalence over 20% or a T/T genotype prevalence over 4%. Several dog breeds showed a moderate variant allele prevalence (10 to 20%), including Shetland Sheepdog (19%), Newfoundland (19%), English Bulldog (18%), French Bulldog (12%), Anatolian Shepherd (10%), Cane Corso (10%). T/T genotype prevalence in these breeds ranged from 1 to 4%. Of the remaining 41 dog breeds evaluated, 20 breeds displayed a low variant allele prevalence (1 to 6%), while the variant allele was not detected in 22 breeds. Mixed-breed dogs showed a relatively low variant allele prevalence (5%).

Interestingly the *SERPINF2* c.605 T allele was also detected in gray wolf populations. Out of 27 animals surveyed, 3 were heterozygous C/T and 2 were homozygous T/T resulting in a variant allele prevalence of 13%.

### 3.3 Functional effect of the *SERPINF2* c.605 C > T amino acid substitution (p.Ala202Val)

Computational analysis by Polyphen-2 (PMID: 20354512) predicted that the p.Ala202Val substitution was possibly damaging with a score of 0.569 (sensitivity 0.88; specificity 0.91). PolyPhen-2 scores can range from 0.0 to 1.0 with higher scores considered more disruptive to protein function.

## 4 Discussion

The results of this study confirm that DEPOH occurs in at least one sighthound breed other than Greyhound and Scottish Deerhound (i.e., Irish Wolfhound). This finding is consistent with anecdotal reports of DEPOH by Irish Wolfhound owners and breeders^4^. The clinical presentation of DEPOH in this case was similar to previous reports for Greyhounds (1, 3, 4) and Scottish Deerhounds (8). The initial sign was peri-incisional bruising, which was first noted 3 days after routine abdominal surgery and continued to spread over the torso. Unfortunately, the dog rapidly succumbed 24 h later before any diagnostic tests or treatment could be initiated.

Although deaths have been described previously in dogs with DEPOH, this is the first reported case to receive a necropsy. This examination confirmed the extensive cutaneous hemorrhage that extended into the subcutaneous tissues. There was also evidence for extensive nonspecific tissue hemorrhage throughout the abdominal and thoracic cavity. Some free and clotted blood was found in the abdomen. However, the source of this blood was unclear since all surgical ligatures and sutures were intact. These findings are consistent with signs of hyperfibrinolysis, although no measurement of fibrinolysis rate was performed. Like all 3 Scottish Deerhounds reported to have died from DEPOH in a previous study (8), this case was homozygous variant *SERPINF2* c.605 T/T genotype thereby implicating a role for alpha-2 antiplasmin deficiency or dysfunction in the pathophysiology of this case. Interestingly when 7 months old, this case also experienced persistent bleeding for 1 week following an ear laceration. This unexpectedly prolonged bleeding may have been related to the same underlying pathophysiology.

The prevalence of the *SERPINF2* c.605 T allele has not yet been reported for any breed other than Scottish Deerhound. Greyhounds, which were the first breed reported with DEPOH, showed the highest prevalence of all breeds surveyed. Interestingly, the population prevalence for the high risk *SERPINF2* c.605 T/T genotype in Greyhounds was 29%, which is within the frequency range of Greyhounds affected by DEPOH (26 to 30%) previously observed in several prospective studies following routine gonadectomy (3, 4). The second highest *SERPINF2* c.605 T allele prevalence was observed in Irish Wolfhounds, the dog breed of this case. As expected, most sighthound breeds showed a moderate to high *SERPINF2* c.605 T allele prevalence of at least 10%. However, the *SERPINF2* c.605 T allele was not detected in 3 sighthound breeds evaluated, including Afghan, Pharoah Hound, and Silken Windhound. The reason for the high prevalence in most sighthound breeds is unclear but may be a consequence of selective breeding for (or against) traits associated with the *SERPINF2* c.605 T allele.

Six of the 47 non-sighthound dog breeds surveyed also showed a moderate *SERPINF2* c.605 T allele frequency (10 to 20%). Phylogenetic analysis has shown that the sighthound breeds cluster into 2 distinct genetic clades (12). Interestingly, 4 of these non-sighthound breeds (Shetland Sheepdog, English Bulldog, French Bulldog, and Cane Corso) are most closely related to the larger sighthound genetic clade containing Greyhound, Irish Wolfhound, Scottish Deerhound, Italian Greyhound, Whippet, and Borzoi. Anatolian Shepherd (10% allele frequency) also clusters closely with the smaller sighthound genetic clade containing Saluki, Ibizan Hound, Afghan Hound, and Pharoah Hound. Conversely, Newfoundland (19% allele frequency) does not cluster with any of the sighthound breeds.

These genotyping results for non-sighthound breeds indicate that up to 4% of certain breeds (such as Newfoundland, Cane Corso, and Boxer) could be at risk for developing DEPOH after surgery as a consequence of having the *SERPINF2* c.605 T/T genotype. However, so far DEPOH has not yet been reported in dog breeds other than sighthounds. There are several possible reasons for this disparity. The most likely explanation is that, until now, DEPOH has been assumed to be a sighthound specific disorder and any unexpected postoperative bleeding or bruising episodes attributed to another cause. The results from this study may encourage veterinarians with cases in non-sighthound breeds that have experienced signs consistent with DEPOH to test for the *SERPINF2* c.605 C > T variant after excluding other bleeding disorders. Additional retrospective and prospective studies are needed to determine the incidence and severity of DEPOH in dogs with the *SERPINF2* c.605 T/T genotype.

The *SERPINF2* c.605 T allele was also found in 5 of 27 unrelated gray wolves. This finding indicates that the *SERPINF2* c.605 T allele may have arisen prior to canine domestication and breed divergence. Interestingly, the basenji showed the third highest *SERPINF2* c.605 T allele frequency (47%). The Basenji is an ancient breed of hunting dog derived from Africa that is considered a basal member of the domestic dog clade (14). This observation also supports the early origin of the *SERPINF2* c.605 T allele prior to breed divergence.

Polyphen-2 computational analysis predicted that the p.Ala202Val protein change associated with the *SERPINF2* c.605 T/T genotype was possibly disruptive to alpha-2 antiplasmin function. Interestingly the Polyphen-2 score determined here (0.569) is almost twice the score (0.314) for the same amino acid change reported previously (8). The reason for the difference in score between studies is likely related to periodic updates in comparative protein sequence alignment and structural data that the Polyphen-2 server receives from various sources^5^. Regardless, these results do not predict complete loss of alpha-2 antiplasmin function in dogs with the *SERPINF2* c.605 T/T genotype. Additional studies are needed to understand the association between this *SERPINF2* variant and increased risk for postoperative bleeding.

There are several limitations to the current study. Hyperfibrinolysis is hypothesized to underly the observed delayed bleeding in this case caused by alpha-2 antiplasmin deficiency. Fibrinolysis rate was not measured. Methods to sensitively and accurately measure fibrinolysis such as thromboelastography (TEG) require fresh whole blood samples and instruments are generally restricted to tertiary care facilities. Newer point-of-care whole blood viscoelastic monitoring devices may provide a solution, although protocol modifications are needed to measure fibrinolysis rate, which must be standardized. Alpha-2 antiplasmin assays are available and can be used on stored plasma samples. A previous study showed reduced median alpha-2 antiplasmin activities in Greyhounds that had experienced DEPOH after surgery compared with those that did not (4). However, activities for most dogs remained within the reference range and there was considerable overlap between DEPOH-susceptible and DEPOH-resistant dogs. Consequently, alpha-2 antiplasmin deficiency in affected dogs is likely partial, not complete, and alpha-2 antiplasmin activities cannot differentiate affected from unaffected dogs.

## 5 Conclusion

When taken together, the results of this study suggest that DEPOH associated with the *SERPINF2* c.605 C > T variant could occur in most, but not all sighthound dog breeds. Furthermore, some non-sighthound dog breeds may be at risk for DEPOH. Further research is needed to obtain better estimates of the susceptibility of different breeds to DEPOH, especially in those breeds with a relatively high prevalence of the *SERPINF2* c.605 C > T variant.

## Data Availability

Publicly available datasets were analyzed in this study. These data can be found here: https://www.ncbi.nlm.nih.gov/bioproject/PRJNA448733/.

## Footnotes

^1^https://www.akc.org/sports/coursing/lure-coursing/; accessed 23 January 2025.

^2^http://genetics.bwh.harvard.edu/pph2/; accessed 20th July 2025.

^3^https://www.ncbi.nlm.nih.gov/home/proteins/; accessed 20th July 2025.

^4^https://www.iwclubofamerica.org/post-operative-bleeding; accessed 6 March, 2025.

^5^ http://genetics.bwh.harvard.edu/wiki/!pph2/faq
