# Supplementary material for: Delayed postoperative hemorrhage (DEPOH) in an Irish Wolfhound with the SERPINF2 c.605 T/T genotype: case description and variant prevalence across dog breeds
Source: Front Vet Sci. 2025 Nov 6;12:1609780. doi: 10.3389/fvets.2025.1609780 (PMC12631970; doi:10.3389/fvets.2025.1609780)
Supplement: Supplementary file 1 [file Table_1.DOCX]

Supplemental Table 1. Number (N) and percentage (%) of dogs with each *SERPINF2* c.605 C>T genotype and observed minor allele frequencies (%) determined for 64 different owner designated dog breeds, mixed breed dogs, and gray wolves. Data were obtained by genotyping DNA samples from the WSU DNA bank or extracted from a public database (Plassais et al, 2019) with at least 20 individuals sampled per breed group.

| Breed | *SERPINF2* c.605 C>T genotype [N (%)] | | | | | |  | Minor Allele Frequency (%) |
| --- | --- | --- | --- | --- | --- | --- | --- | --- |
|  | C/C | | C/T | | T/T | | N dogs |  |
| Afghan | 36 | (100) | 0 | (0) | 0 | (0) | 36 | 0.0 |
| Alaskan Malamute | 39 | (95) | 2 | (5) | 0 | (0) | 41 | 2.4 |
| American Staffordshire Terrier | 19 | (90) | 2 | (10) | 0 | (0) | 21 | 4.8 |
| Anatolian Shepherd | 29 | (81) | 7 | (19) | 0 | (0) | 36 | 9.7 |
| Australian Cattle Dog | 53 | (93) | 4 | (7) | 0 | (0) | 57 | 3.5 |
| Australian Shepherd | 52 | (95) | 3 | (5) | 0 | (0) | 55 | 2.7 |
| Basenji | 10 | (26) | 20 | (51) | 9 | (23) | 39 | 48.7 |
| Beagle | 55 | (96) | 1 | (2) | 1 | (2) | 57 | 2.6 |
| Bernese Mountain Dog | 46 | (100) | 0 | (0) | 0 | (0) | 46 | 0.0 |
| Border Collie | 78 | (88) | 11 | (12) | 0 | (0) | 89 | 6.2 |
| Borzoi | 70 | (90) | 8 | (10) | 0 | (0) | 78 | 5.1 |
| Boston Terrier | 53 | (93) | 4 | (7) | 0 | (0) | 57 | 3.5 |
| Boxer | 52 | (93) | 2 | (4) | 2 | (4) | 56 | 5.4 |
| Brittany Spaniel | 27 | (100) | 0 | (0) | 0 | (0) | 27 | 0.0 |
| Cane Corso | 21 | (84) | 3 | (12) | 1 | (4) | 25 | 10.0 |
| Cardigan Welsh Corgi | 22 | (100) | 0 | (0) | 0 | (0) | 22 | 0.0 |
| Cavalier King Charles Spaniel | 27 | (100) | 0 | (0) | 0 | (0) | 27 | 0.0 |
| Chihuahua | 23 | (100) | 0 | (0) | 0 | (0) | 23 | 0.0 |
| Chow Chow | 50 | (98) | 1 | (2) | 0 | (0) | 51 | 1.0 |
| Cocker Spaniel | 32 | (100) | 0 | (0) | 0 | (0) | 32 | 0.0 |
| Collie | 28 | (97) | 1 | (3) | 0 | (0) | 29 | 1.7 |
| Dachshund | 25 | (100) | 0 | (0) | 0 | (0) | 25 | 0.0 |
| Doberman Pinscher | 41 | (100) | 0 | (0) | 0 | (0) | 41 | 0.0 |
| English Bulldog | 39 | (68) | 16 | (28) | 2 | (4) | 57 | 17.5 |
| French Bulldog | 47 | (78) | 12 | (20) | 1 | (2) | 60 | 11.7 |
| Galgo Español | 14 | (45) | 13 | (42) | 4 | (13) | 31 | 33.9 |
| German Shepherd | 51 | (88) | 7 | (12) | 0 | (0) | 58 | 6.0 |
| Golden Retriever | 87 | (92) | 5 | (5) | 3 | (3) | 95 | 5.8 |
| Great Dane | 75 | (93) | 5 | (6) | 1 | (1) | 81 | 4.3 |
| Grey Wolf | 22 | (81) | 3 | (11) | 2 | (7) | 27 | 13.0 |
| Greyhound | 164 | (25) | 300 | (46) | 185 | (29) | 649 | 51.6 |
| Ibizan Hound | 30 | (97) | 1 | (3) | 0 | (0) | 31 | 1.6 |
| Irish Wolfhound | 20 | (23) | 45 | (52) | 21 | (24) | 86 | 50.6 |
| Italian Greyhound | 29 | (38) | 32 | (42) | 16 | (21) | 77 | 41.6 |
| Labrador Retriever | 83 | (97) | 3 | (3) | 0 | (0) | 86 | 1.7 |
| Miniature Dachshund | 20 | (100) | 0 | (0) | 0 | (0) | 20 | 0.0 |
| Miniature Poodle | 22 | (100) | 0 | (0) | 0 | (0) | 22 | 0.0 |
| Miniature Schnauzer | 22 | (96) | 1 | (4) | 0 | (0) | 23 | 2.2 |
| Mixed breed | 170 | (91) | 13 | (7) | 3 | (2) | 186 | 5.1 |
| Newfoundland | 36 | (67) | 16 | (30) | 2 | (4) | 54 | 18.5 |
| Old English Sheepdog | 22 | (100) | 0 | (0) | 0 | (0) | 22 | 0.0 |
| Pembroke Welsh Corgi | 24 | (100) | 0 | (0) | 0 | (0) | 24 | 0.0 |
| Peruvian Inca Orchid | 16 | (76) | 4 | (19) | 1 | (5) | 21 | 14.3 |
| Pharaoh Hound | 25 | (100) | 0 | (0) | 0 | (0) | 25 | 0.0 |
| Pitbull | 33 | (92) | 3 | (8) | 0 | (0) | 36 | 4.2 |
| Pitbull | 42 | (93) | 3 | (7) | 0 | (0) | 45 | 3.3 |
| Pomeranian | 21 | (100) | 0 | (0) | 0 | (0) | 21 | 0.0 |
| Pug | 25 | (100) | 0 | (0) | 0 | (0) | 25 | 0.0 |
| Rhodesian Ridgeback | 39 | (95) | 2 | (5) | 0 | (0) | 41 | 2.4 |
| Rottweiler | 51 | (98) | 1 | (2) | 0 | (0) | 52 | 1.0 |
| Saint Bernard | 25 | (100) | 0 | (0) | 0 | (0) | 25 | 0.0 |
| Saluki | 92 | (50) | 72 | (39) | 19 | (10) | 183 | 30.1 |
| Samoyed | 24 | (100) | 0 | (0) | 0 | (0) | 24 | 0.0 |
| Scottish Deerhound | 392 | (60) | 221 | (34) | 42 | (6) | 655 | 23.3 |
| Shetland Sheepdog | 47 | (63) | 27 | (36) | 1 | (1) | 75 | 19.3 |
| Shih Tzu | 21 | (100) | 0 | (0) | 0 | (0) | 21 | 0.0 |
| Siberian Husky | 25 | (100) | 0 | (0) | 0 | (0) | 25 | 0.0 |
| Silken Windhound | 68 | (100) | 0 | (0) | 0 | (0) | 68 | 0.0 |
| Soft-coated Wheaten Terrier | 30 | (100) | 0 | (0) | 0 | (0) | 30 | 0.0 |
| Springer Spaniel | 21 | (95) | 1 | (5) | 0 | (0) | 22 | 2.3 |
| Standard Poodle | 25 | (100) | 0 | (0) | 0 | (0) | 25 | 0.0 |
| Toy Poodle | 20 | (100) | 0 | (0) | 0 | (0) | 20 | 0.0 |
| Weimaraner | 22 | (100) | 0 | (0) | 0 | (0) | 22 | 0.0 |
| Whippet | 99 | (59) | 55 | (33) | 14 | (8) | 168 | 24.7 |
| Windsprite | 28 | (93) | 2 | (7) | 0 | (0) | 30 | 3.3 |
| Yorkshire Terrier | 98 | (94) | 5 | (5) | 1 | (1) | 104 | 3.4 |
